# Trade-offs in the externalities of pig production are not inevitable

**DOI:** 10.1038/s43016-024-00921-2

**Published:** 2024-04-11

**Authors:** Harriet Bartlett, Márcia Zanella, Beatriz Kaori, Leandro Sabei, Michelle S. Araujo, Tauana Maria de Paula, Adroaldo J. Zanella, Mark A. Holmes, James L. N. Wood, Andrew Balmford

**Affiliations:** 1 Department of Zoology, University of Cambridge, Cambridge, UK; 2 Department of Veterinary Medicine, University of Cambridge, Cambridge, UK; 3 Smith School of Enterprise and Environment, University of Oxford, Oxford, UK; 4 Department of Biology, University of Oxford, Oxford, UK; 5 Department of Preventive Veterinary Medicine and Animal Health, School of Veterinary Medicine and Animal Science, University of São Paulo, São Paulo, Brazil

**Keywords:** Agroecology, Climate-change mitigation, Antibiotics, Environmental impact, Agriculture

## Abstract

Farming externalities are believed to co-vary negatively, yet trade-offs have rarely been quantified systematically. Here we present data from UK and Brazilian pig production systems representative of most commercial systems across the world ranging from ‘intensive’ indoor systems through to extensive free range, Organic and woodland systems to explore co-variation among four major externality costs. We found that no specific farming type was consistently associated with good performance across all domains. Generally, systems with low land use have low greenhouse gas emissions but high antimicrobial use and poor animal welfare, and vice versa. Some individual systems performed well in all domains but were not exclusive to any particular type of farming system. Our findings suggest that trade-offs may be avoidable if mitigation focuses on lowering impacts within system types rather than simply changing types of farming.

## Abstract (web summary)

Greenhouse gas emissions, antimicrobial use, land use and animal welfare data representing most global commercial pig production systems show that no single system performs well across all measures, but trade-offs may be avoidable if mitigation measures are implemented within farming systems.

## Main

Livestock farming generates major impacts. While it provides 30% of human dietary protein and 18% of calories^1^, it occupies 75% of agricultural land^2^, emits 14–17% of anthropogenic greenhouse gas (GHG) emissions^3,4^ and uses more antimicrobials than human medicine^5^. Livestock production is rapidly growing^6^, especially pig production, which has quadrupled in the past 50 years (www.fao.org/faostat). Externalities of farming—consequences that affect external parties—are thought to trade off with one another, with types of farm that perform well in one domain performing consistently poorly in others^7–13^. However, different externalities are typically examined in isolation, and associations among them have only been empirically and systematically quantified for narrow sets of systems and externalities^9,14,15^. The few studies that consider how externalities co-vary across contrasting production systems find trade-offs are less common than typically perceived. For example, a negative association was found between freshwater use and GHGs and between freshwater use and eutrophication in tomato systems, although GHGs and eutrophication had a positive association^16^. Positive associations have been shown between GHG and soil organic carbon costs in Chinese grain^17^ and among GHGs, acidification and eutrophication in Iranian wheat and barley^18^. However, only a small subset have been carried out in livestock systems. These have reported positive associations among land-use, GHG, nitrogen, phosphorus and soil costs for European dairy; between land-use and GHG costs for Brazilian beef^9^; and between GHG, acidification, eutrophication, non-renewable energy and land-use costs for European beef^19^. Reference ^20^ found positive associations between GHG, land use, water use and fossil fuel costs in Brazilian beef but trade-offs among these costs with metal depletion and acidification costs. One other study^21^ examined animal welfare and antimicrobial use (AMU) costs and found no association in Italian dairy farms. Importantly, none has included both environmental costs and animal welfare^9,22–24^. Without understanding the associations among externalities, it is impossible to provide informed guidance about reducing the negative effects of farming^8^. Currently, for some externalities the impacts of different practices are frequently assumed, and in some cases effectively ignored, with potentially counterproductive consequences.

In this Article, we examine four critically important externalities that have never been systematically measured together across any farming system. Our analysis provides empirical data allowing the systematic comparison of many alternative farming systems and their consequences for land use (as an externality in its own right and as a proxy for biodiversity loss^25^), GHG emissions, AMU (as a proxy for antimicrobial resistance) and animal welfare. We focus on pig production because pork is already the second most commonly eaten meat worldwide by mass, global pork production has grown at 1.2% yr^−1^ since 2005 (www.fao.org/faostat), and this growth is predicted to continue through to 2050 (ref. ^26^). We focus on four externality costs where urgent mitigation is needed and where pig production imposes substantial burdens: land use, because pig production already uses 8.5% of arable land^27^; GHG emissions, because pig production accounts for 9% of GHGs from livestock^4^; AMU as pig production uses more antimicrobials than any other livestock sector^28^ and the livestock sector uses twice as much as human medicine^29^; and welfare because pigs are sentient and intelligent^30^. Our large primary dataset has allowed us to examine all four of our focal externalities. Our study farms spanned most of the world’s commercial pig farm types, and our data represent the annual production of over 1.2 million pigs. Our objectives were to quantify four externalities of central importance to society (land use^25^, GHG emissions^31^, AMU^32^ and animal welfare^33^) across a broad range of contrasting farms, to test whether these externalities trade off against each other and to identify those best- and worst-performing systems and types of system.

## Results

### Externality cost estimates

Each of our 74 UK and 17 Brazilian data points corresponded to a real-world, commercial breed-to-finish system made up of one to three farms. Each externality was aggregated across the whole lifecycle of production (see Methods for further details) expressed per functional unit (per kilogram deadweight (DW)) and, for clarity, is referred to as a cost where a high value indicates a more harmful outcome.

**Fig. 1:**
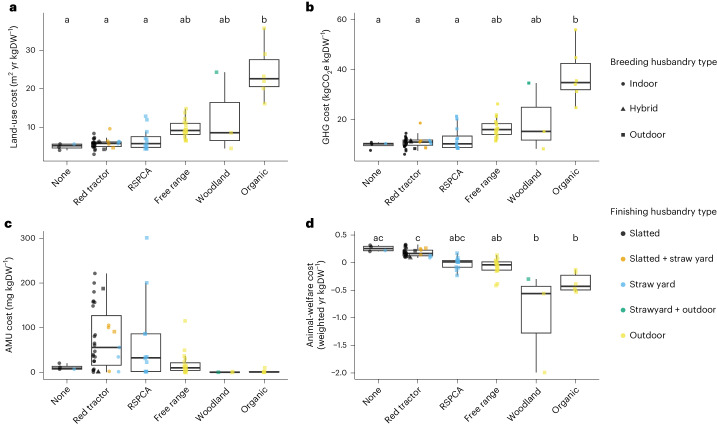
The externality costs of 74 UK breed-to-finish pig systems by label type. Three assurance schemes (Organic, the welfare-focused RSPCA assured and traceability-focused Red tractor) as well as two other non-assurance product labels, free range and woodland. **a**, Land-use costs, including land to rear pigs and to grow their feed. **b**, GHG costs, including emissions associated with feed production, enteric fermentation, manure management, energy, fuel, transport, slaughter, processing and following recent assessments^9,53,54,64,70,71^ also included forgone sequestration—the carbon opportunity cost which accounts for the difference in carbon stored in farmed versus non-farmed land. **c**, Total AMU costs (for data on AMU of greatest importance to human health^24^, see Extended Data Fig. 2). **d**, Animal-welfare costs using methods set out in ref. ^34^, which weighted the quantity of life-years required to produce 1 kg DW by quality-of-life scores based on detailed animal-based welfare assessments we carried out for each system (Methods). The shapes and colours of scattered points show the husbandry type of breeding and finishing subsystems, respectively. Letters above boxplots show Dunn’s post hoc results, controlled for multiple comparisons using the Holm method, with different letters indicating significant differences (*P* < 0.05). Analyses were carried out on a subset of our data (*n* = 43) with one datapoint selected randomly from those that shared breeding and/or rearing herds (Methods). Upper and lower hinges correspond to the first and third quartiles. Upper and lower whiskers extend to 1.5 times the interquartile range from upper and lower hinges, respectively. The middle horizontal bar is the median.

Land-use costs across both UK and Brazilian systems varied by a factor of 12 from 3.0 to 35.8 m^2^ yr kgDW^−1^, and GHG costs varied by a factor of 9 from 6.2 to 55.9 kgCO_2_e kgDW^−1^ (1.3 to 12.2 kgCO_2_e kgDW^−1^ if excluding forgone sequestration; Fig. 1). We used two AMU metrics which are both reported in mg kgDW^−1^: (1) total use, which ranged from 0 to 606 mg kgDW^−1^, and (2) use of those critically important to human health^24^ (Methods) which ranged from 0 to 65.7 mg kgDW^−1^ (Fig. 1). Applying a previously established metric^34^, we find that animal-welfare costs ranged widely, with some systems assessed as harmful to welfare and others where the quality of life was deemed sufficiently high that they were assessed as being beneficial (Fig. 1).

**Extended Data Fig. 1:**
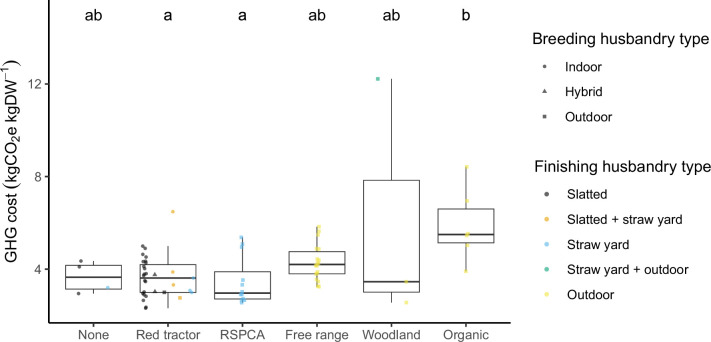
GHG costs of 74 UK breed-to-finish pig systems by label type excluding foregone sequestration. GHG costs varied significantly among label types (Kruskal-Wallis: χ^2^ = 16.0, df=5, p < 0.01, n = 43), with Organic costs being significantly higher than RSPCA assured and Red tractor (post-hoc Dunn’s analysis p = 0.02 and p = 0.01 respectively). Letters above boxplots show the results from Dunn’s post-hoc tests, controlled for multiple comparisons using the Holm method, with different letters indicating significant differences between median values. Upper and lower hinges correspond to the first and third quartiles. Upper and lower whiskers extend to 1.5 times the interquartile range from upper and lower hinges respectively. The middle horizontal bar is the median.

**Extended Data Fig. 2:**
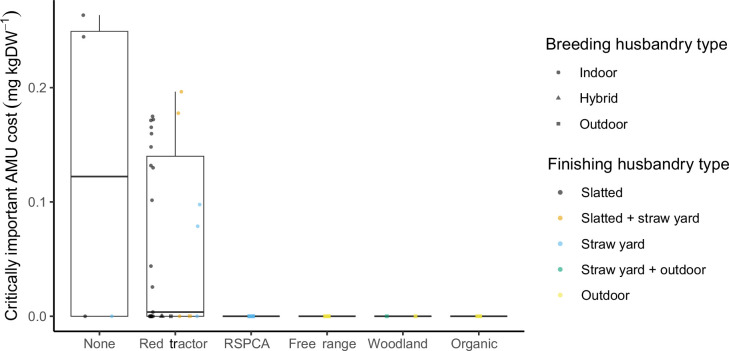
Critically important AMU costs of 74 UK breed-to-finish pig systems by label type. Critically important AMU costs did not vary significantly by label type (Kruskal-Wallis p = 0.1; n = 43). Upper and lower hinges correspond to the first and third quartiles. Upper and lower whiskers extend to 1.5 times the interquartile range from upper and lower hinges respectively. The middle horizontal bar is the median.

**Extended Data Fig. 3:**
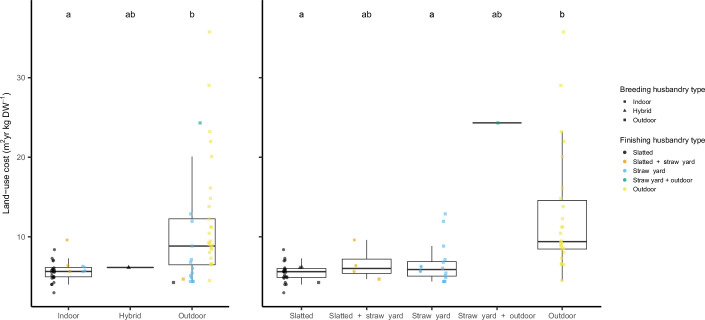
Land-use costs of 74 UK breed-to-finish pig systems by a) breeding and b) finishing husbandry type. This included land to rear animals and produce feed. The shapes and colours of scattered points show the husbandry type of breeding and finishing subsystems respectively. There were significant differences in land-use costs among husbandry types (Kruskal-Wallis: χ^2^ = 11.1, df=2 and χ^2^ = 23.3, df=4 for breeding and finishing system respectively, both p < 0.01, n = 43). Letters above boxplots show the results from Dunn’s post-hoc tests, controlled for multiple comparisons using the Holm method, with different letters indicating significant differences between median values. Indoor-bred systems had lower land-use costs than outdoor bred (p < 0.01), and outdoor-finished than straw yard (p = 0.01) and slatted (p < 0.01). Upper and lower hinges correspond to the first and third quartiles. Upper and lower whiskers extend to 1.5 times the interquartile range from upper and lower hinges respectively. The middle horizontal bar is the median.

**Extended Data Fig. 4:**
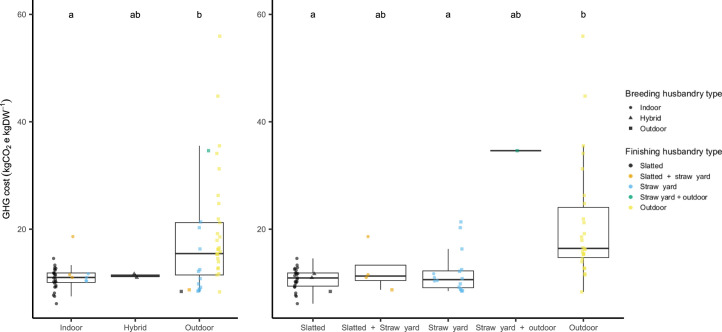
GHG costs of 74 UK breed-to-finish pig systems by a) breeding and b) finishing husbandry type. GHG cost included animal source emissions, those associated with feed production, transport, energy use, slaughter, processing and forgone sequestration. The shapes and colours of scattered points show the husbandry type of breeding and finishing subsystems respectively. There were significant differences in GHG costs among husbandry types (Kruskal-Wallis: χ^2^ *=* 9.2, df=2, p *=* 0.01 and χ^2^ *=* 18.4, df=4, p *>* 0.01 for breeding and finishing system respectively, both n *=* 43). Letters above boxplots show the results from Dunn’s post-hoc tests, controlled for multiple comparisons using the Holm method, with different letters indicating significant differences between median values. Indoor-bred systems had lower GHG costs than outdoor bred (p *<* 0.01), and outdoor-finished than straw yard and slatted (both p *<* 0.01). Upper and lower whiskers extend to 1.5 times the interquartile range from upper and lower hinges respectively. The middle horizontal bar is the median.

**Extended Data Fig. 5:**
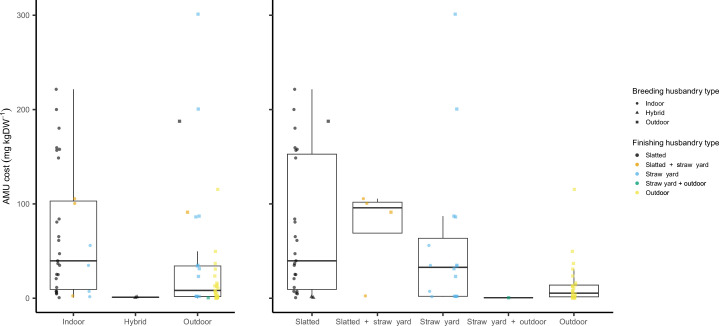
AMU costs of 74 UK breed-to-finish pig systems by a) breeding and b) finishing husbandry type. There were significant differences between breeding husbandry types (χ^2^ *=* 7.31, df=2, p *=* 0.03, n *=* 43) but not finishing husbandry types (Kruskal-Wallis p *=* 0.1, n *=* 43). Post-hoc Dunn’s analyses did not find any pairs with significant differences. Upper and lower hinges correspond to the first and third quartiles. Upper and lower whiskers extend to 1.5 times the interquartile range from upper and lower hinges respectively. The middle horizontal bar is the median.

**Extended Data Fig. 6:**
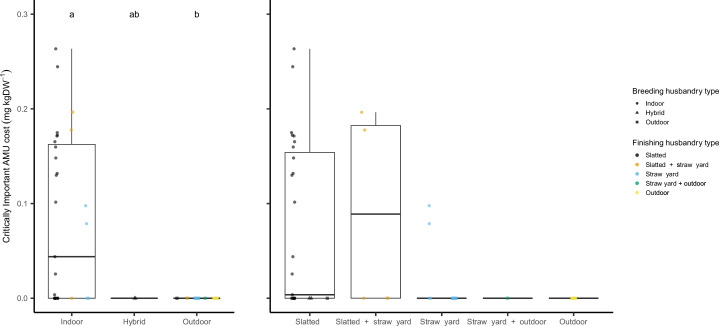
Critically important AMU costs of 74 UK breed-to-finish pig systems by a) breeding and b) finishing husbandry type. There were significant differences between breeding husbandry types (χ^2^ = 10.9, df=2, p < 0.01, n = 43) but not finishing husbandry types (Kruskal-Wallis p = 0.1, n = 43). Post-hoc Dunn’s analyses found that critically important AMU cost was higher in indoor-bred systems compared with outdoor-bred systems. Letters above boxplots show the results from Dunn’s post-hoc tests, controlled for multiple comparisons using the Holm method, with different letters indicating significant differences between median values (p > 0.05). Upper and lower hinges correspond to the first and third quartiles. Upper and lower whiskers extend to 1.5 times the interquartile range from upper and lower hinges respectively. The middle horizontal bar is the median.

**Extended Data Fig. 7:**
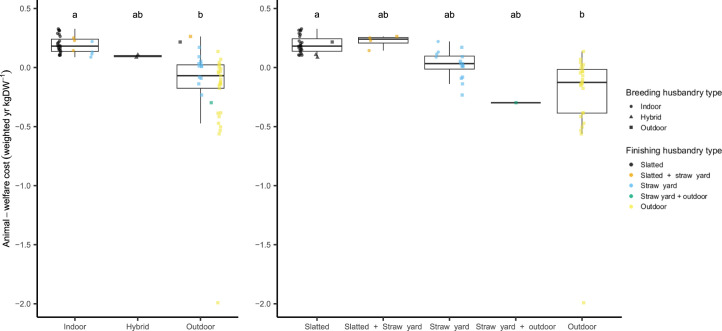
Animal welfare costs of 74 UK breed-to-finish pig systems by a) breeding and b) finishing husbandry type. There were significant differences among costs by husbandry type (Kruskal-Wallis by breeding type χ^2^ = 32.1, df=2, p < 0.01 and husbandry type χ^2^ = 31.4, df=4, p < 0.01, both n = 43). Post-hoc Dunn’s analyses found indoor-bred systems had higher costs than outdoor-bred systems (p < 0.01), and slatted-finished systems had higher costs than outdoor-finished (p > 0.01). Letters above boxplots show the results from Dunn’s post-hoc tests, controlled for multiple comparisons using the Holm method, with different letters indicating significant differences between median values. Upper and lower hinges correspond to the first and third quartiles. Upper and lower whiskers extend to 1.5 times the interquartile range from upper and lower hinges respectively. The middle horizontal bar is the median.

We did not find significant differences in land-use and GHG costs between UK and Brazilian systems (Wilcoxon rank-sum test, *P* > 0.2), although this may be due to the small sample of Brazilian farms. Brazilian systems had significantly higher total and critically important AMU costs and animal-welfare costs than UK systems (Wilcoxon rank-sum test, all *P* < 0.01). In UK systems there were significant differences among label types in land-use costs (Kruskal–Wallis *χ*^2^ = 23.3, d.f. = 5, *P* < 0.01) with Organic costs higher than the Royal Society for the Prevention of Cruelty to Animals (RSPCA) assured, Red tractor and ‘none’ (post hoc Dunn’s analyses all *P* < 0.01). There were also significant differences in GHG costs (*χ*^2^ = 21.9, d.f. = 5, *P* < 0.01) with Organic costs higher than RSPCA assured, Red tractor and ‘none’ (post hoc Dunn’s analyses *P* < 0.01, *P* > 0.01 and *P* = 0.02, respectively; see Extended Data Fig. 1 for results excluding forgone sequestration), and total AMU costs (Kruskal–Wallis *χ*^2^ = 11.7, d.f. = 5, *P* = 0.04; note that no pairwise comparisons were significant—all *P* > 0.2—and that critically important AMU costs did not differ among label types; Extended Data Fig. 2). There were significant differences among label types for animal-welfare costs (Kruskal–Wallis *χ*^2^ = 34.5, d.f. = 5, *P* < 0.01) with greater costs in ‘none’ and Red tractor systems than in woodland and Organic (post hoc Dunn’s *P* = 0.01, *P* < 0.01 and both *P* < 0.01, respectively), and in Red tractor than free range (*P* = 0.01). There were also significant differences in some costs among husbandry types (Extended Data Figs. 3–7).

### UK externality costs with positive associations

**Fig. 2:**
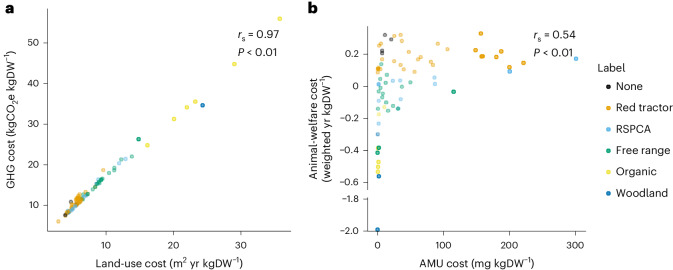
Positive associations among externality cost across 74 UK pig production systems. **a**,**b**, Land-use and GHG costs (**a**) and AMU and animal welfare costs^34^ (**b**), with higher externality costs representing increasingly negative outcomes. Note the break in the animal-welfare cost axis due to the very low cost of one system. *r*_s_ and *P* values were from two-sided Spearman rank correlations on a subset of our data (*n* = 43) with one datapoint selected randomly from those that shared breeding and/or rearing herds (Methods).

**Extended Data Fig. 8:**
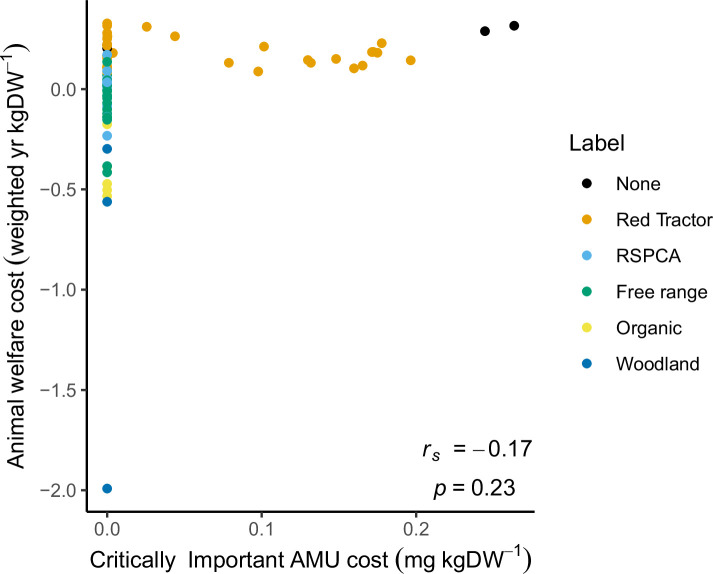
Critically important AMU and welfare costs of UK breed-to-finish pig systems. Critically important AMU is just use of those antimicrobials most important to human health – Category B use according to the European Medicines Agency. r_s_ and p values are from two-sided Spearman rank correlations on a subset of our data (n = 43) with one datapoint selected randomly from those that shared breeding and/or rearing herds (see Methods).

Land-use and GHG costs of contrasting UK pig production systems were strongly positively associated with one another (Fig. 2a). This association remained when GHG costs excluded forgone sequestration (Spearman rank *r*_s_ = 0.84, *P* < 0.01; Supplementary Fig. 1). Animal-welfare costs were moderately positively associated with AMU costs (Fig. 2b), and we found no significant association between animal-welfare costs and critically important AMU costs (Extended Data Fig. 8).

### UK externality costs with negative associations

**Fig. 3:**
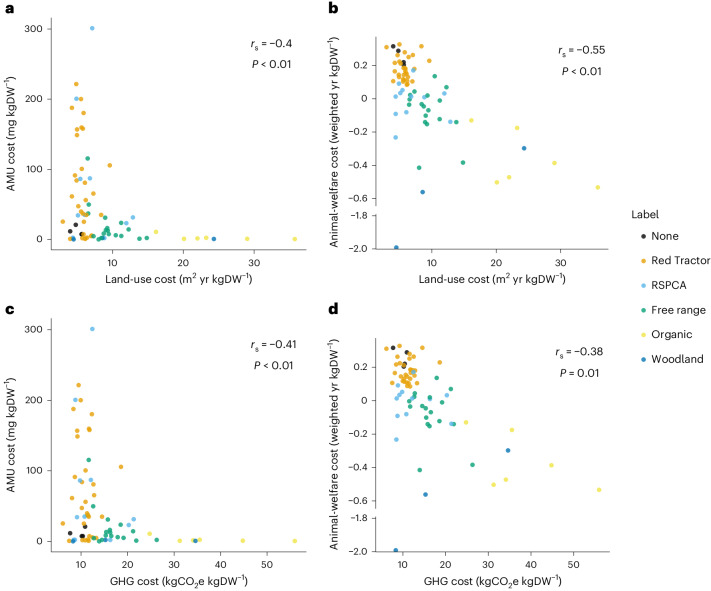
Negative associations among externality costs across 74 UK pig production systems. **a**–**d**, Associations between land-use cost and total AMU cost (**a**), between land-use cost and animal-welfare cost (**b**) between GHG cost and total AMU cost (**c**) and between GHG cost and animal-welfare cost (**d**) across 74 UK pig systems. *r*_s_ and *P* values were from two-sided Spearman rank correlations on a subset of our data (*n* = 43) with one datapoint selected randomly from those that shared breeding and/or rearing herds. Note that there are breaks in the animal-welfare cost axes due to the very low costs of one system.

In contrast to these positive associations, AMU and animal welfare both had moderate negative associations with both GHG and land-use costs (Fig. 3)—systems that performed well according to one pair of externalities and typically performed poorly in terms of the other pair of measures of impact. Although some associations are not simple negative linear trends (Fig. 3a,c), these patterns suggest that in broad terms there were trade-offs. Pig systems either performed well in land use and GHGs and poorly for animal welfare and AMU, or vice versa. However, it is important to examine not just overall relationships but to look at individual systems as well.

### UK systems with co-benefits

**Fig. 4:**
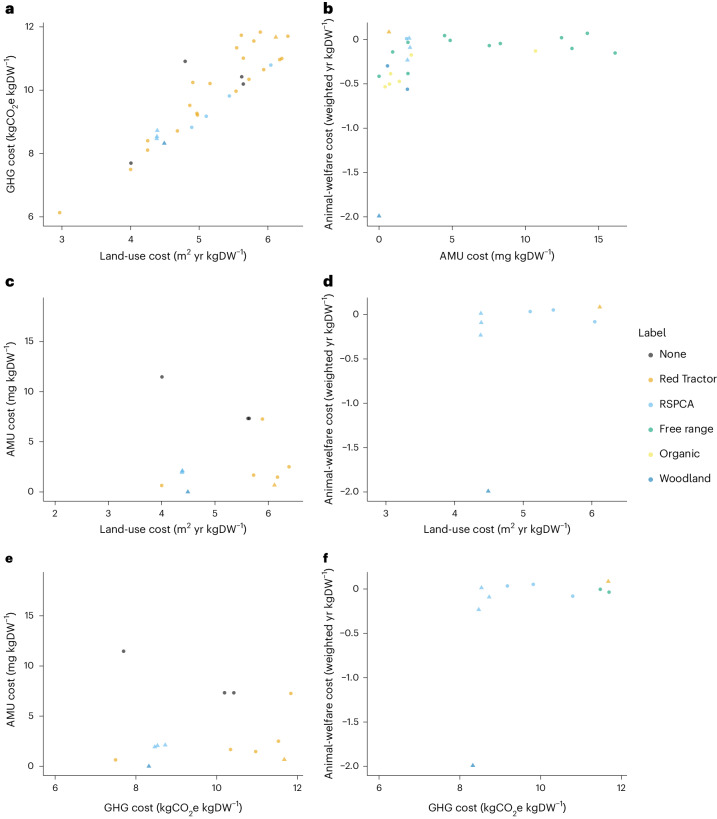
The best-performing systems. **a**–**f**, Each plot shows UK breed-to-finish pig systems in the top 50% for both respective externality costs: land use and GHG (**a**), AMU and animal welfare (**b**), land use and AMU (**c**), land use and animal welfare (**d**), GHG and AMU (**e**), and GHG and animal welfare (**f**). The five systems shown as triangles are those that performed in the best 50% for all four externality costs.

Focusing on those systems ranked in the best-performing 50% of our sample for each cost, we can see that these broad trade-offs are not inevitable. For each pair of negatively associated externalities, several systems did not fit the overall negative association and instead combined low costs in both externalities (Fig. 4). Importantly, the best-performing systems overall were spread across different types of pig farming, but no type performed consistently well. Of the five systems in the best-performing 50% for all four externalities (shown as triangles in Fig. 4), three were RSPCA assured systems (of 27 in this study) that were outdoor bred and straw-yard finished, one was a fully outdoor woodland system (of three assessed here) and one was a Red tractor system (of 61) with hybrid indoor–outdoor breeding and slatted finishing. This list of consistently best-performing systems did not change if forgone sequestration was not included in our estimate of GHG costs. A further 10 systems were in the best-performing 50% by three externality costs: these ranged from indoor-bred, slatted-finishing systems with no labelling through to outdoor-bred, straw-yard finished RSPCA assured systems; in this case excluding forgone sequestration from GHG costs did slightly alter which systems met this threshold (Supplementary Table 1). Of these, six did not meet the benchmark of being in the best 50% for welfare cost, three did not meet the AMU cost benchmark and one system did not meet the GHG cost benchmark. It is noteworthy that no Organic nor free-range systems appeared in the best-performing 50% in three or more domains as none was in the best 50% for either land-use nor GHG cost. However, 100% of the six Organic systems and 61% of 18 free-range systems were in the best-performing 50% for both welfare and AMU, as were all three woodland systems. One system was in the best-performing 25% for all externality costs: an RSPCA assured system with outdoor breeding and straw-yard finishing.

**Extended Data Fig. 9:**
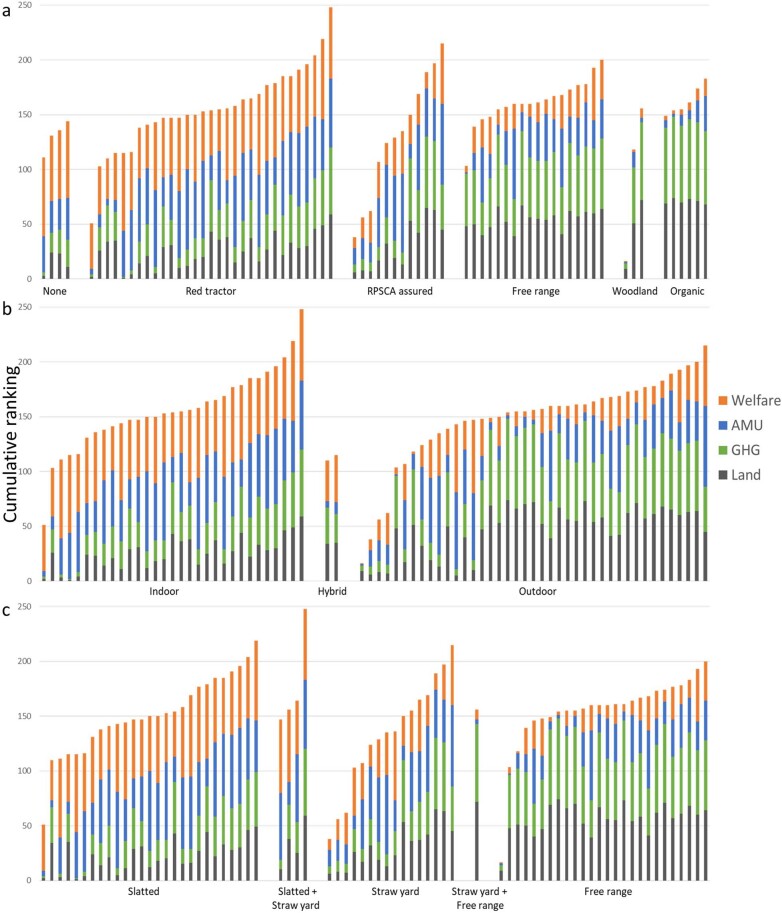
The summed ranking of each of 74 UK breed-to-finish system by a) label type, and b) breeding system and (c) finishing system. Each system was given an externality cost ranking from 1 for the best performing system to 74 for worst performing system, and these were summed across the four externalities. If a system was best in all four costs it would have a score of 4, and 296 if worst in all.

All label and husbandry types that had systems in the best-performing 50% also had systems that were in the worst-performing half in at least one domain (Extended Data Fig. 9). Four systems were in the bottom 50% for all four externalities: three Red tractor systems and one RSPCA assured system; one Red tractor system was in the bottom performing 25% for all externalities. No type was consistently associated with low costs across our four domains, which has important implications for consumers as labelling does not allow them to make informed decisions about these externalities.

### Examination of Brazilian pig production

**Fig. 5:**
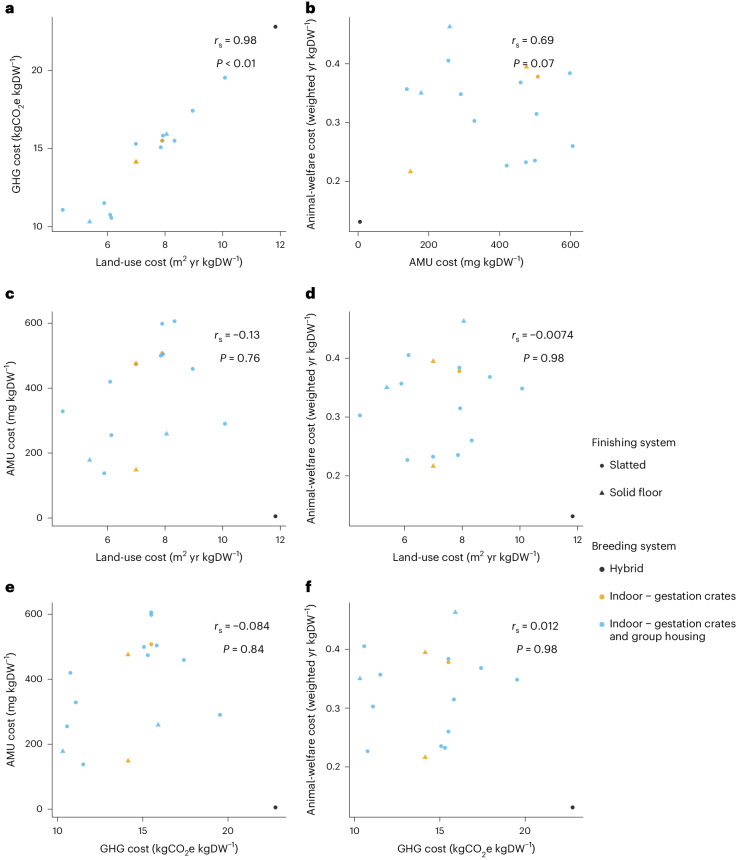
Externality costs of 17 Brazilian pig systems. **a**–**f**, The associations among land-use and GHG costs (**a**), AMU and animal-welfare costs (**b**), land-use and AMU costs (**c**), land-use and animal-welfare costs (**d**), GHG and AMU costs (**e**) and GHG and animal-welfare costs (**f**). *r*_s_ and *P* values are from two-sided Spearman rank correlations on a subset of our data (*n* = 8) with one data point selected randomly from those that shared breeding and/or rearing herds. Our sample was too small to identify significant differences among husbandry types.

These broad insights from UK systems were generally echoed when comparing 17 Brazilian pig systems. We found a significant positive association between land-use cost and GHG cost (which again remained when forgone sequestration was excluded; Supplementary Fig. 2) and some evidence of a positive association between AMU and animal-welfare cost (Fig. 5; note that colours refer to husbandry types as there are no established labels in Brazil). We found less clear associations than in the UK between other pairs of externalities, which may be due to our smaller sample size. However, once again we found some systems—in this case three—that were in the top 50% for all four externality costs and a further five in the top 50% for three costs. These results suggest that, as in the UK, trade-offs among contrasting externality costs are not consistent in Brazilian pig production and that there are systems that perform above average across all four of the costs we measured.

### The costs of switching pig systems at scale

The consequences of changes in farm types can be illustrated by considering the hypothetical externality costs of meeting all of current UK pig production through a single system. Meeting 2021 UK pig production (based on Agriculture and Horticulture Development Board figures; https://ahdb.org.uk/uk-pig-facts-and-figures) entirely via Organic production, based on its median externality costs from this study, would result in considerably less AMU (88–98% less, an overall reduction of 8–56 tonnes of active ingredient per year) and substantial welfare improvements, compared with production from entirely Red tractor, RSPCA assured systems or systems with no label type. However, Organic systems would have three times as much impact on the climate as Red tractor, RSPCA assured systems or systems with no label type (an additional 25 million tonnes of CO_2_ equivalents per year; 2.6 million tonnes excluding forgone sequestration) and use four times as much land (an additional 1.7 million hectares per year; ~10% of UK agricultural land).

## Discussion

The exceptional scale of impacts of global agriculture, and particularly livestock production, makes it imperative to search for farming systems that impose low externality costs. We tested the associations, across different methods of livestock production, of key externalities which are commonly perceived to trade off with one another. We found evidence that while trade-offs do indeed exist, they were not inevitable. Comparing systems that span most commercial pig production found across the world, we find that systems with low land use typically have low GHG costs but high AMU costs and poor welfare. This finding aligns with common perceptions^35–38^, which were until now based on assumption. However, this overall view masks the equally important finding that some systems achieve low externality costs across all domains. Notably, we find no unique characteristics among the best- or worst-performing systems and that no label or husbandry type is a reliable indicator of neither these best- nor worst-performing systems.

Previous studies focus on externalities in isolation and have small sample sizes, but when adjusted for functional units and system boundaries, our results for individual externalities align well for land-use^39^, GHG^23,40^, AMU^28,41^ and animal-welfare costs^42^.

How different groups of stakeholders prioritize, value and balance different externalities would determine which farm and label types would result in the best outcomes. Our findings suggest that mitigation should not simply focus on changing broad types of farming but must also include lowering impacts within system types. Within each farming type, we find there is substantial scope for improvement—for example, land-use costs range by more than a factor of 5 within woodland systems. Disappointingly, we find that our current ways of classifying UK pig systems (and hence marketing pork) are not identifying and promoting the best-performing systems for land use, the climate, AMU and animal welfare. This means our current labelling systems are not helping consumers or indeed regulators to identify systems that perform well across multiple areas of societal concern. We suggest that interventions may be more effective if they focus on measurable improvements in outcomes rather than changes in farm types.

Our analyses may be limited by the size of our sample of farms, their geographic range and their spread across farm types. Despite the large and diverse sample of pig production systems covered by our dataset, we were still only able to recruit, for example, three commercial woodland farms. Nevertheless, our sample was sufficient to identify promising systems that perform better than average in terms of each of land-use, GHG, AMU and animal welfare costs. We can also be confident in our identification that free range and Organic pig production rarely if ever perform well across all four domains, as our study had very high coverage of these sectors (60% and 47% of UK production, respectively; Methods) and no farm we examined from either group was in the top 50% for land-use or GHG costs. This finding is in contrast to common perceptions of these types of system^43^.

Our findings are also inevitably limited by data quality. Due to strict regulations in the UK on record keeping (of AMU, livestock transport, feed use and so on), we are confident that our UK AMU data are reasonably accurate. In Brazil, the quality of antimicrobial record keeping was more variable. Fortunately, excluding those systems with poorer-quality records did not alter our conclusions (Supplementary Fig. 3). Other sources of uncertainty are likely in both countries—from imputing missing data (Methods), from our choice of crop land and GHG footprint data, from our choice of emissions factors, because of non-reporting of unfinished antimicrobials (which probably led to slight over-estimation of AMU costs) and because of the snapshot approach of our welfare assessments. In addition, our land-use cost estimates did not account for differences in the impacts of land use on biodiversity (except that under tree cover; see Methods for explanation) as it was only possible to obtain sourcing information at the level of country of origin for many feed crops, and methods for comparing biodiversity impacts of agricultural products are rudimentary and limited in their regional specificity^44^. It will be important to account for these differences in future analyses when robust methods for equating the biodiversity opportunity costs of land use are available.

We are limited by the number of externalities we could practically examine. Future work should consider and incorporate additional environmental externalities important in pig production such as eutrophication and acidification^45^. It also would be valuable to expand the scope of analyses such as this to consider broader social outcomes, such as the effects of pork consumption on public health, the financial viability and scalability of contrasting production systems, and their implications for the well-being of farmers.

In summary, we suggest that our results confirm that in seeking to increase the sustainability of agriculture, it is not enough to assume relationships between externalities or even simply to look at general trends based on high-quality data. We need instead to consider individual farms, identify those that appear best at limiting externality costs across a broad range of outcomes of societal concern, and understand, promote and incentivize their practices. We hope that the present work encourages others to undertake similar but complementary studies, covering more externalities and, critically, other important but poorly documented agricultural sectors. We believe such analyses are essential for enabling the identification and promotion of the most promising options for mitigating the impacts of food production systems.

## Methods

### Sampling and classification of farms

**Extended Data Fig. 10:**
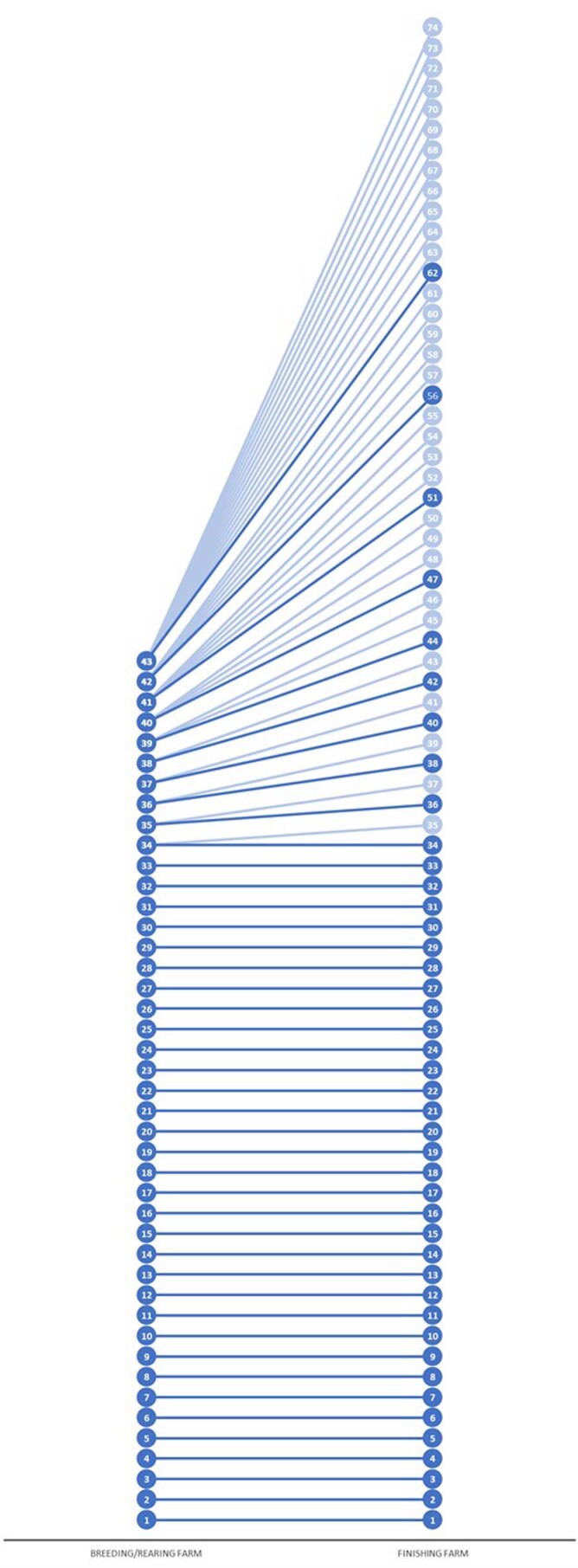
Diagram of which UK finishing and fattening farms shared breeding or rearing herds. 33 breed-to-finish systems did not share breeding/rearing herds with another – shown by single horizontal lines from breeding/rearing farm to finishing farms 1-33. 41 breed-to-finish systems did share breeding and/or rearing herds – shown by the multiple lines from breeding/rearing farm on the left leading to the multiple finishing farms 34-74 on the right. In our statistical analysis, one finishing farm was randomly selected from those that shared breeding and/or rearing farms – as illustrated by the different shades of blue: farms included in statistical analyses are shown in dark blue and those excluded in pale blue.

We contacted 150 UK and 30 Brazilian pig producers, of which 44 and 20 producers participated in this study, respectively. We contacted farmers with the help of collaborating industry professionals, internet searches and social media. Sample bias was minimized by actively recruiting farm types that might otherwise be underrepresented with the help of experts. Our dataset consisted of breed-to-finish systems, which each involved one to three farms (see Extended Data Fig. 10 for a visualization of this), and some producers had more than one farm. Our final dataset consisted of 74 UK and 17 Brazilian data points—each a breed-to-finish system, with a unique finishing or fattening farm, but some shared breeding and/or rearing farms, so our data points are not all independent. We accounted for this in our analysis (‘Statistical analyses’).

We classified UK systems by labelling type (taken here as membership of the quality assurance schemes Red tractor, RSPCA assured and Organic, and two other non-assurance labels—free range and woodland; and by husbandry type, how the pigs are housed by breeding and finishing system; Supplementary Table 2). The Brazilian pig sector does not have such well-established assurance schemes but has globally important husbandry types not present in the UK such as systems with gestation crates^46^ and that use growth promoters (for example, ractopamine). We therefore classified Brazilian systems only by husbandry type (Supplementary Table 3).

### Data collection and allocation

Each farm was visited between September 2017 and December 2020. We conducted welfare assessments and questionnaire-based interviews with farm managers to collect data for simultaneously estimating land-use, GHG, AMU and animal-welfare costs for the most recent year of available data. Where upstream farms did not exclusively supply one finishing or fattening farm, externality costs were allocated proportionately by the number of pigs entering and leaving each farm. For example, if a breeding farm produced 1,000 weaned piglets and 500 of these moved to the finishing or fattening farm in question, 50% of the costs of the breeding farm were allocated to the finishing or fattening farm.

### Externality cost quantification

The system boundaries (Supplementary Methods) included feed production, pig production from breeding to finishing, slaughter and processing. All externalities were measured per functional unit (per kilogram DW) which is key to comparing the costs of producing a given amount of food in different ways^9^. The kg DW was calculated using data collected via the interview on productivity, animal numbers and either DW output or liveweight and dressing percentages. DW included meat from finishing pigs and sows sent to slaughter, which were equated with economic allocation using mean prices between September 2018 and December 2020 from the UK Agriculture and Horticulture Development Board (https://ahdb.org.uk/pork/gb-deadweight-pig-prices-uk-spec) and a large British pork processor (anonymous, personal communication) for UK systems. Similar data were not available for Brazilian pig production, so this same allocation was used. See Supplementary Methods for equations used to calculate externality costs.

### Land-use cost

Land-use cost was the total area of land required over 1 year to produce 1 kg DW of pork in m^2^ yr kgDW^−1^ and includes land to rear pigs and grow their feed. Field studies examining the density of >2,000 wild species of vertebrates, flowering plants and insects on five continents have found that farming would have least impact on biodiversity if high-yield production was coupled with sparing remaining land for nature^9^. We, therefore, considered land use not just as a direct environmental impact in itself but also as a proxy for impact on biodiversity, with low land-use costs (high yields) representing lower impacts on biodiversity.

#### Land-use cost to rear pigs

The area of land used to rear pigs was obtained via the questionnaire or calculated from a map. Pigs kept in woodland are argued to have a positive impact on biodiversity^47,48^, although there is limited empirical evidence to support this. Therefore, we cautiously assumed this land to have equal biodiversity value to natural habitat without pigs, and so excluded land used to rear pigs under tree cover from our land-use costs. The observation that one of our woodland systems was in the best 50% for all externalities was dependent on this assumption—when land under woodland was not excluded, it was no longer in the top 50% for land-use cost.

#### Land-use cost to grow feed

The area of land used to grow feed was calculated using farm- and production-stage specific feed formulations (which cannot be shared due to Intellectual Property constraints) and the total amount of each feed used in the most recent year. For the small portion of feeds for which we could not obtain precise formulations (1.5% by mass for UK systems and 1.1% for Brazilian systems), the most similar formulation was used instead. Feed ingredient yields were obtained from FeedPrint^49^, using country-specific data where possible. Where the country of origin was unknown or unavailable, the most similar feed or country with available data was used. Some feed formulations reported aggregated rather than individual ingredients. These were again gap-filled using information from the most similar feeds. For example, some feeds had added synthetic amino acids, but the formulation was not broken down into specific amino acids. In this case, feed amino acid contents were matched to the total amino acid levels of a similar feed using data from feedtables.com. For organic feed ingredient yields, we applied percentage differences in conventional versus organic yields for wheat, barley, oats and beans from ref. ^50^, maize from ref. ^51^ and soya and peas from ref. ^52^. Where feeds were co-products, economic allocation was used to assign costs to the focal feed using data from FeedPrint.

### GHG cost

GHG cost was the mass of CO_2_e (in kilograms) emitted in the production of 1 kg DW. This included emissions from feed production (including fertilizer manufacture), the pigs, fuel, energy, transport, slaughter and meat processing and also forgone sequestration (the carbon opportunity cost of land use^9,53,54^). Methane and nitrous oxide were converted into CO_2_e using GWP100.

#### GHG cost from feed

Feed emissions included those associated with feed ingredient production, milling and processing, and were obtained via Feedprint, using country-specific values where possible. Where there were gaps in our data, they were filled as with land-use costs. GHG emissions associated with producing organic feeds were calculated by applying percentage differences in GHG footprints of conventional versus organic GHGs using data from Williams et al.^55^. No Brazilian farms used organic feeds.

#### GHG cost from pigs

Emissions from pigs themselves included enteric methane, and nitrous oxide and methane from manure management. Enteric manure methane was calculated using emissions factors from https://naei.beis.gov.uk/data/ef-all for the UK and Intergovernmental Panel on Climate Change tier 1 emissions factors for Brazil^56^. Manure methane was calculated using the gross energy intake we calculated using farm and production-stage specific feeds (using data on gross energy of feed ingredients from feedtables.org) and activity data and emissions factors from ref. ^56^. Direct and indirect nitrous oxide emissions were calculated from nitrogen input (which we calculated using farm and production stage-specific feeds) using nitrogen content of feed from feedtables.org and nitrogen utilization rates from ref. ^57^. Residual manure nitrogen was assumed to displace fertilizer use in feed production as in Weidema et al.^58^ (Ecoinvent v3. vol. 3; http://www.ecoinvent.org/, 2014). GHG costs were insensitive to contrasting approaches to accounting for displaced fertilizer (see Supplementary Fig. 4 for a sensitivity analysis).

#### GHG cost from fuel, electricity and transport

Annual use of fuel and electricity were converted into kg CO_2_ using emissions factors from ref. ^59^ and www.carbonfootprint.com. Emissions from travel were calculated for a return journey per tonne-km transported using emissions factors from ref. ^60^ for Brazil and ref. ^61^ for the UK. Where farm-specific data were available (that is, producers reported the location of the feed mill/crops) these were used. If only the region (municipality, state) was known, the centroid of this was taken. Where regions were unknown, we used the country- and crop-specific mean distance travelled for other farms in this study that did not grow their own feed. It was not possible to gain information for transport upstream of feed farms—that is, from crop farms to feed mills—so FeedPrint values were used. This includes transport emissions associated with manure spreading on feed crops.

#### GHG cost from slaughter and processing

Emissions from slaughter and meat processing were assumed to be the same as in ref. ^62^.

#### GHG cost from forgone sequestration

As has been explored in several recent papers, farming carries an additional GHG cost—that of forgone sequestration, because the carbon stored in farmed land is less than that of native habitat that could in principle occupy that land in the absence of farming^9,54^. Forgone sequestration was calculated using values for annual aboveground biomass accrual (<20 years) taken from ref. ^56^, assuming the relevant native habitat in the area used for production. In the UK this was ‘Temperate Oceanic Forest—Europe’, except for sorghum and soybean products which were assumed to be ‘Tropical Moist Deciduous Forest—North and South American’. For Brazilian systems, we used accrual rates for ‘Tropical Moist Deciduous Forest—North and South America’. Changes in soil carbon were taken from the mean percentage change in soil organic carbon from crop to secondary forest from ref. ^63^. Initial carbon values were taken from ref. ^56^, and changes were assumed to take 20 years, following Intergovernmental Panel on Climate Change guidelines. We assumed soils in the UK were ‘cold temperate, moist, high activity soils’ except for sorghum and soybean products which were assumed to be ‘subtropical, humid, low activity soils’, and in Brazil were ‘subtropical, humid, low activity soils’. See Extended Data Fig. 1 and Supplementary Figs. 1 and 2 for GHG costs excluding forgone sequestration. Our results for GHG costs were broadly insensitive to contrasting approaches to accounting for forgone sequestration (see Supplementary Fig. 5 for a sensitivity analysis testing the effect of halving sequestration rates)^64^.

### AMU cost

AMU cost was the milligrams of antimicrobial used to produce 1 kg DW. AMU cost was calculated from medicines records for the most recent year of available data obtained via the questionnaire. Reference ^24^ found it was important to report both the total use and use of critically important antimicrobials for human health because the latter tend to be lightweight and therefore easily obscured by total use metrics. We focus on total use metrics to enable comparison with other studies as these are the most commonly used metrics. However, we also report critically important use as use of category B antimicrobials according to the European Medicines Agency (Extended Data Figs. 2, 6 and 8).

### Animal-welfare cost

We estimated the animal-welfare cost of each pig production system as the number of life years required to produce 1 kg DW weighted by quality-of-life scores—calculated in the same way as ref. ^34^, a study we conducted to identify LCA-compatible animal welfare metrics which built upon WQ (Welfare Quality) assessments. Ref. ^34^ was based on UK animal welfare data from this dataset; here, we extend the metric to all systems and combine it with the other indicators mentioned above. H.B. (who is WQ certified) conducted assessments for breeding pigs and pre-weaning piglets and for fattening pigs for each of our data points. WQ assessments involve the systematic measurement of more than 30 different welfare indicators for each system, including a range of indicators of health and behaviour. Protocols were carried out according to guidelines except where sows were kept in crates for part or all of gestation. During the time sows were kept in crates, as there are no opportunities for social interactions, the worst possible score for social behaviour was given. WQ assessments result in four principle scores out of 100, for good health, good feeding, good housing and appropriate behaviour. There is no consensus on how these principles should be weighted when combined into an overall score. Reference ^34^ found system rankings were very little affected by the weighting approach, so we used a simple weighting of 0.35, 0.25, 0.15 and 0.25, respectively. The assessments for sows and piglets and for fattening pigs were combined by multiplying the quantities of life years required by each to produce 1 kg DW by their respective WQ scores and then summing them. There are a broad range of attitudes on what criteria determine good welfare^65–68^—which could mean welfare is viewed in some cases as an externality benefit rather than cost. Reference ^34^ found the relative performance of label types and husbandry types was very largely insensitive to the choice of transition from costly to beneficial welfare, so here we chose a metric with an intermediate approach where WQ principle scores of ≥80 are deemed to be beneficial to welfare, and so time experiencing them was treated as a benefit (negative cost) and lower WQ principle scores as costly.

### Statistical analyses

Some of our data points were not independent of one another as they shared breeding or rearing farms. There were insufficient data to remove the effects of this statistically, so where statistics are reported, this is for a subset of our data (UK, *n* = 43; Brazil, *n* = 8) with one datapoint randomly selected from those that share breeding and/or rearing farms. We used Spearman rank correlations to characterize the associations between externality costs, and to compare farm types we used Wilcoxon rank-sum tests and Kruskal–Wallis tests with post hoc Dunn’s analysis using the Holm method to control for multiple comparisons. Analyses were carried out in RStudio4.1.1 using the packages ‘stats’, ‘FSA’, ‘g’gpubr’, ‘rcompanion’, ‘ggthemes’, ‘patchwork’ and ‘ggplot2’.

### Ethics

Ethical approval was given by the Human Biology Research Ethics Committee (application number 2018.22) at the University of Cambridge and by Plataforma Brasil before commencement. Before participating in the study, all farmers gave informed consent.

### Reporting summary

Further information on research design is available in the Nature Portfolio Reporting Summary linked to this article.

## Supplementary information

Supplementary Information Supplementary Figs. 1–5, Tables 1–3 and Methods.

Reporting Summary

## Data Availability

Data can be found in Figshare repository at 10.6084/m9.figshare.22760723 (ref. ^69^).

## Code availability

Code is available upon request.
