# Author Correction: Trade-offs in the externalities of pig production are not inevitable

**DOI:** 10.1038/s43016-026-01345-w

**Published:** 2026-04-09

**Authors:** Harriet Bartlett, Márcia Zanella, Beatriz Kaori, Leandro Sabei, Michelle S. Araujo, Tauana Maria de Paula, Adroaldo J. Zanella, Mark A. Holmes, James L. N. Wood, Andrew Balmford

**Affiliations:** 1 Department of Zoology, University of Cambridge, Cambridge, UK; 2 Department of Veterinary Medicine, University of Cambridge, Cambridge, UK; 3 Smith School of Enterprise and Environment, University of Oxford, Oxford, UK; 4 Department of Biology, University of Oxford, Oxford, UK; 5 Department of Preventive Veterinary Medicine and Animal Health, School of Veterinary Medicine and Animal Science, University of São Paulo, São Paulo, Brazil

**Keywords:** Agroecology, Climate-change mitigation, Antibiotics, Environmental impact, Agriculture

Correction to: *Nature Food* 10.1038/s43016-024-00921-2, published online 11 April 2024.

In the version of this article originally published, a paper describing the animal‑welfare metric and methodology used (originally cited as ref. 64) is now cited earlier (ref. 34; Bartlett, H. et al. *Proc. Royal Soc. B* 10.1098/rspb.2023.0120 (2023)), with citation updates in the Fig. 1 legend, second paragraph of Results, and with a slightly modified Methods “Animal welfare cost” section, indicating animal quality-of-life scores were calculated in the same way as ref. 34, based on UK animal welfare data, a metric used to inform all indicators in the paper. The Reporting Summary “Research sample” field has been expanded to note “We previously established an animal welfare metric based on UK farms only [ref. 34]. Here, we used animal welfare data from the same farms in the UK, as well as additional data from farms in Brazil.” The changes have been made in the HTML and PDF versions of the article.

