# Supplementary material for: Trade-offs in the externalities of pig production are not inevitable
Source: Nat Food. 2024 Apr 11;5(4):312–22. doi: 10.1038/s43016-024-00921-2 (PMC11045459; doi:10.1038/s43016-024-00921-2)
Supplement: Supplementary file 1 — Supplementary Figs. 1–5, Tables 1–3 and Methods. [file 43016_2024_921_MOESM1_ESM.pdf]

nature food

Supplementary information https://doi.org/10.1038/s43016-024-00921-2

Trade-offs in the externalities of pig production are not inevitable

In the format provided by the authors and unedited

Tradeoffs in the externalities of pork production are not inevitable: Supplementary Materials

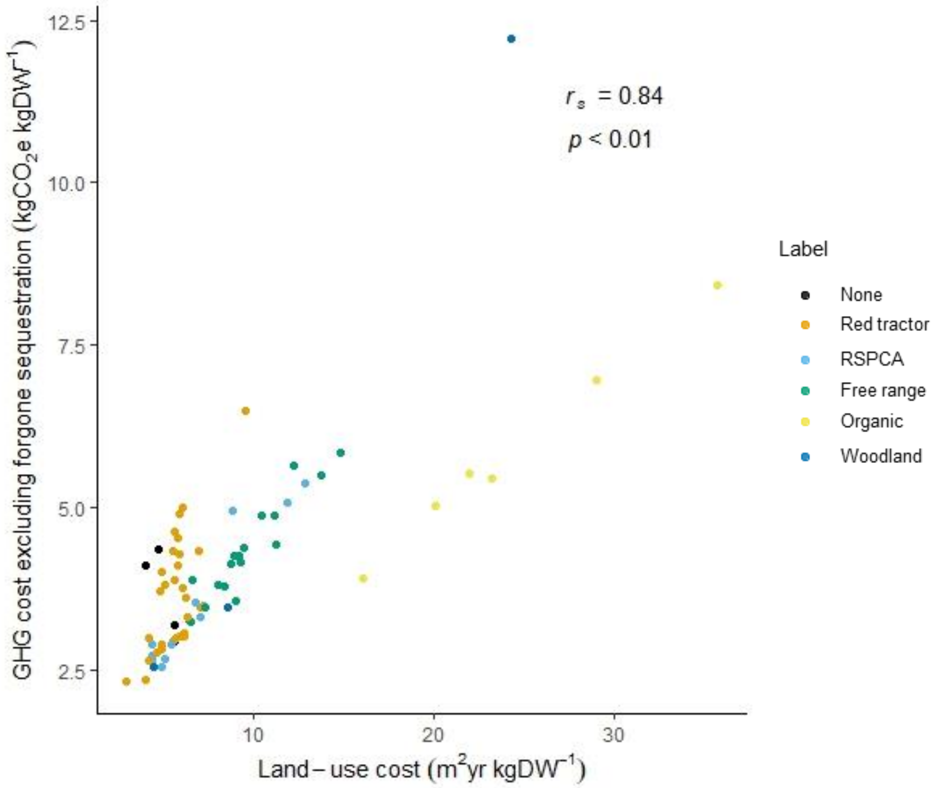

Figure 1 Land-use cost and GHG cost of 74 breed-to-finish UK pig systems. GHG costs here included animal source emissions, those associated with feed production, transport, energy use, slaughter and processing, but excluded foregone sequestration. r_s and p values are from two-sided Spearman rank correlations on a subset of our data (n=43) with one datapoint selected randomly from those that shared breeding and/or rearing herds (see Methods).

Table 1 UK breed-to-finish pig systems in the top performing 50% for three externality costs

**Label type**	**Husbandry type (breeding)**	**Husbandry type (finishing)**	**Number of systems**	**Externality cost system did not meet benchmark for**
None	Indoor	Slatted	2	Welfare
		Straw yard	1	
Red tractor	Hybrid indoor-outdoor	Slatted	1	
	Indoor	Slatted	2	
		Straw yard	1	
RSPCA assured	Outdoor	Straw yard	3	AMU

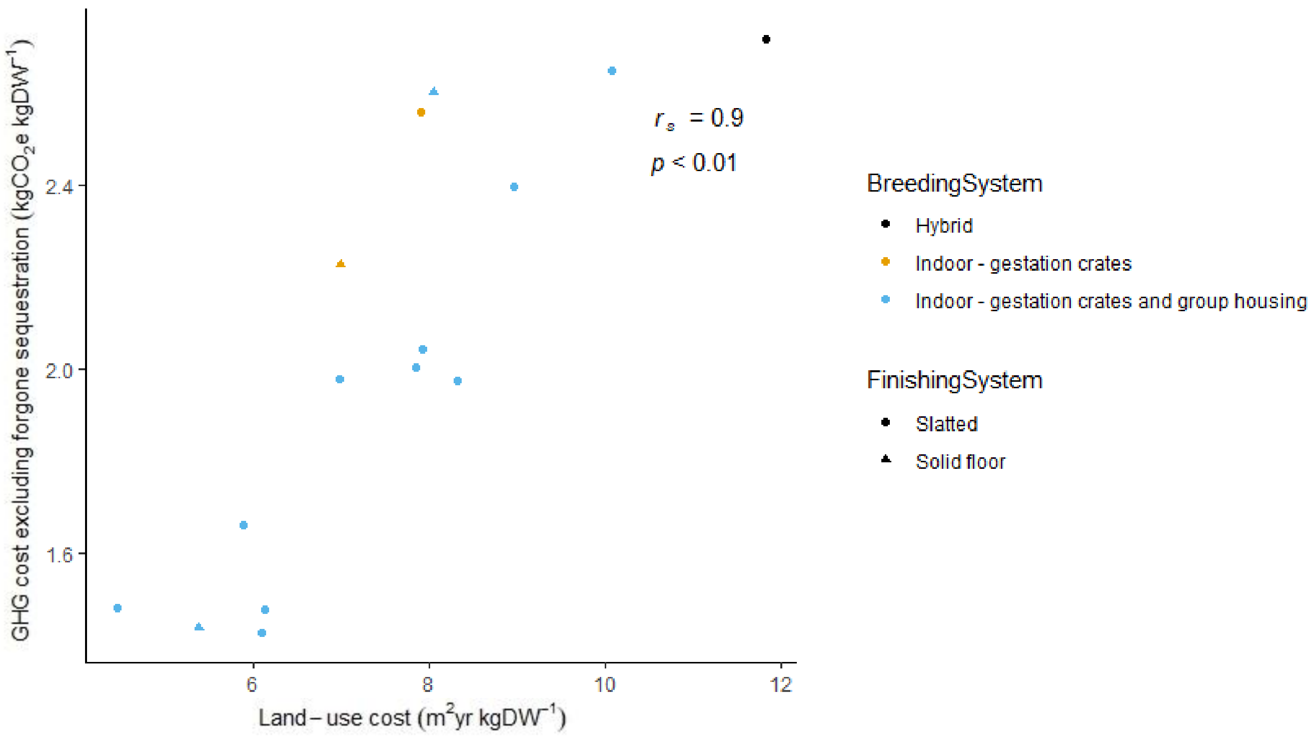

Figure 2 Land-use cost and GHG cost excluding foregone sequestration for 17 Brazilian pig systems. r_s and p values are from two-sided Spearman rank correlations on a subset of our data (n=8) with one datapoint selected randomly from those that shared breeding and/or rearing herds (see Methods).

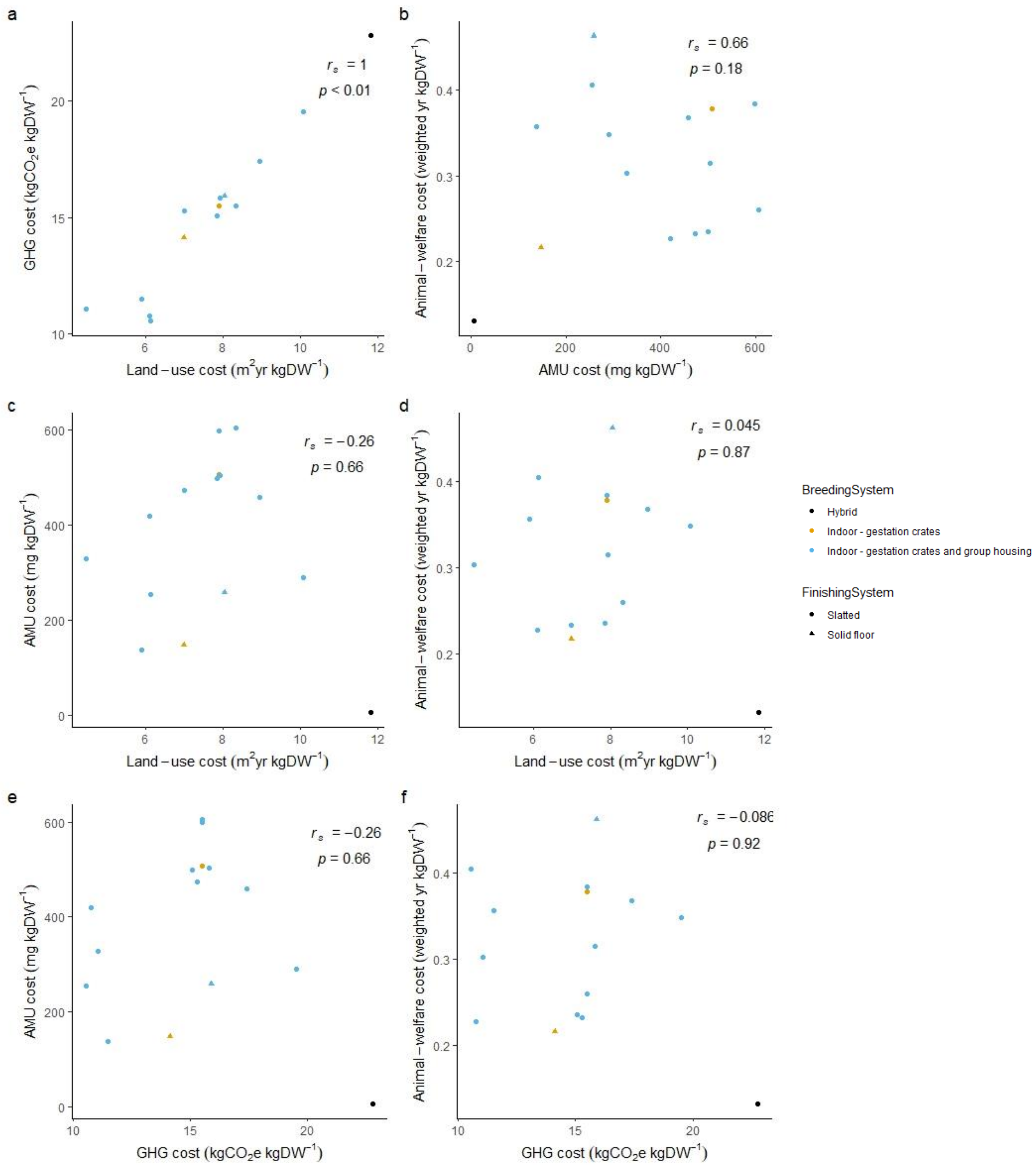

Figure 3 Externality costs of 17 Brazilian pig systems, with two systems with poorer quality data removed. r_s and p values are from two-sided Spearman rank correlations on a subset of our data (n=8) with one datapoint selected randomly from those that shared breeding and/or rearing herds. Our sample was too small to identify significant differences among husbandry types.

Table 2 Description of the 74 breed-to-finish pig systems studied in the UK. The label categories are approximately ordered by the degree of standards required by each, with more demanding categories exceeding the standards of lower categories. From least to most demanding the categories are no assurance or labelling ("None"), Red tractor (including Quality Meat Scotland; QMS), RSPCA assured, free range, woodland and Organic. If systems met the requirements for multiple labels, they were included in the most demanding label type – for example, free range systems that are also RSPCA assured are included in the free range category. Relevant label standards or guidelines can be found at the following websites: Red tractor (www.redtractorassurance.org.uk), QMS (https://qmscotland.co.uk), RSPCA assured (https://science.rspca.org.uk/sciencegroup/farmanimals/standards/pigs) and Organic (www.soilassociation.org/organicstandards and www.orgfoodfed.com). The fifth column shows the percentage of the total slaughtered fattening pigs in the UK in 2021 (from www.gov.uk/government/statistics/cattle-sheep-and-pig-slaughter) that is accounted for by each label type (according to www.pig-world.co.uk/news/highlighting-the-differences-how-uk-welfare-standards-compare-with-our-competitors). These sum to more than 100% as systems often have multiple label types. The final column shows the annual slaughtered fattening pigs from our 74 systems, summed by label type and rounded to the nearest 1,000, and our estimate of the % of all slaughtered pigs belonging to that label type which they represent. In total, our study covers ~5% of UK slaughtered fattening pigs.

Label type	Breeding husbandry type	Rearing and finishing husbandry type	Number of breed-to-finish systems in this study	% UK pigs by label type	Pigs in this study (% of UK total pigs)
**None**	Typically indoors. Farrowing crates are permitted.	Typically indoors. Fully slatted floors are permitted.	4	5	38,000 (7%)
Red tractor* including *QMS	Typically indoors. Farrowing crates are permitted.	Typically indoors. Fully slatted floors are permitted.	31	95	479,000 (5%)
RSPCA assured	Farrowing can be indoors, but sows must be allowed to turn around at all times.	Pigs must have access to unperforated floors and sufficient bedding.	12 (of which 10 are also *Red tractor*)	unknown	222,000 (unknown)
**Free range**	Always outdoors.		18 (of which 15 are also *Red tractor* and *RSPCA assured*)	2.5	165,000 (60%)
**Woodland**	Pigs are kept at least with partial tree cover, but farms could also include some indoor housing.		3 (of which 2 are also free range)	unknown	13,000 (unknown)
Organic	Always outdoors.		6 (of which 5 are also *Red tractor, RSPCA assured* and all 6 are free range)	0.6	31,000 (47%)

Table 3 Description of the 17 breed-to-finish pig systems studied in Brazil, by husbandry type as there are no established labels.

Breeding husbandry type	Rearing and finishing husbandry type	Number of breed-to-finish systems	% Brazilian pigs	Pigs in this study
**Hybrid indoor-outdoor**		1		1,000
**Gestation crates and group housing**	Typically indoors. Fully slatted floors are permitted.	3	0.65	99,000
**Gestation crates only**		13		201,000

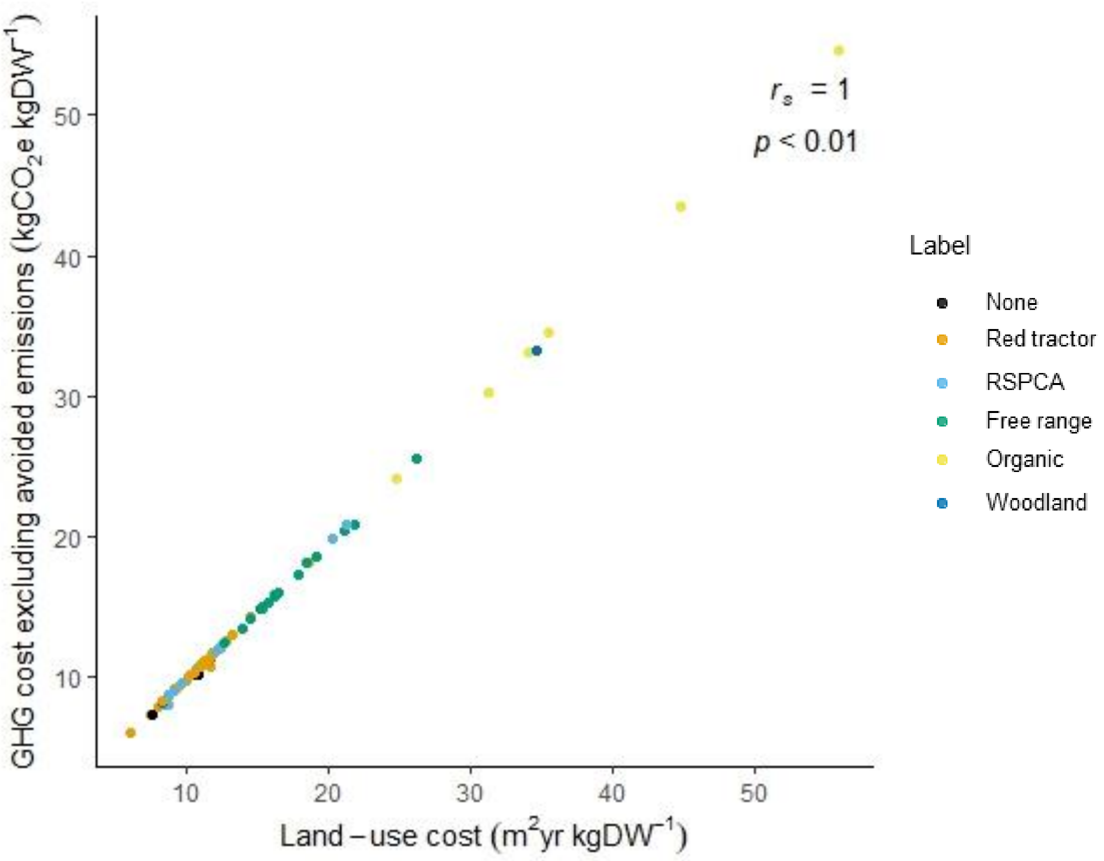

Figure 4 Sensitivity of GHG cost to accounting for avoided emissions due to manure replacing fertiliser on our 74 UK datapoints. GHG cost (x axis) included emissions from animals, energy, fuel, transport, feed production and manure management. This assumed that nitrogen remaining in manure was spread on fields and displaces fertiliser use as in ref.[1]. GHG cost excluding avoided emissions (y axis) assumed manure did not displace any fertiliser use. r_s and p values are from two-sided Spearman rank correlations on a subset of our data (n=43) with one datapoint selected randomly from those that shared breeding and/or rearing herds (see Methods).

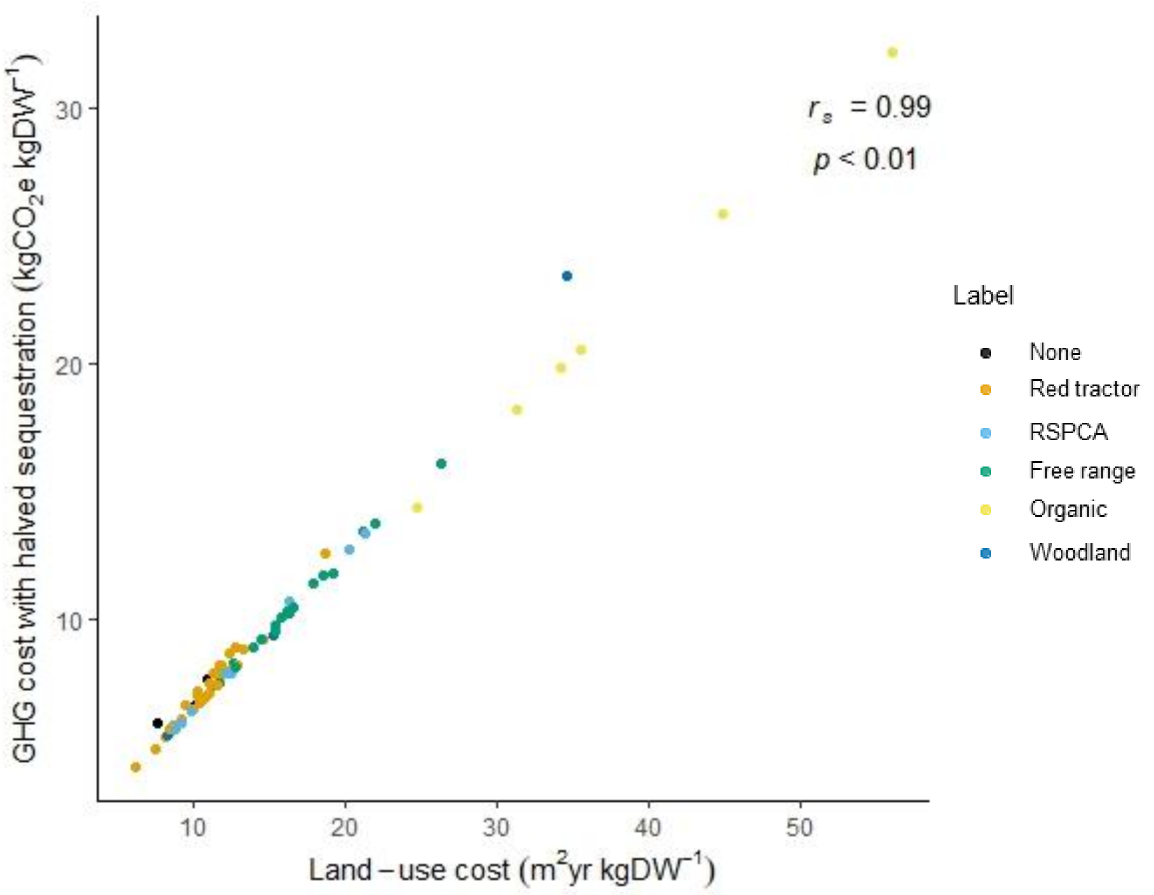

Figure 5 Sensitivity of GHG cost to accounting for forgone sequestration, for our 74 UK datapoints. GHG cost (x axis) included emissions from animals, energy, fuel, transport, feed production, manure management and foregone sequestration (see Methods). GHG cost: half sequestration rates (y axis) assumed that the forgone sequestration rates were half those in GHG cost. r_s and p values are from two-sided Spearman rank correlations on a subset of our data (n=43) with one datapoint selected randomly from those that shared breeding and/or rearing herds (see Methods).

Supplementary Methods

The following sections outline the equations used to calculate externality costs, which were all calculated over the most recent year of available data.

Land-use cost

Land-use cost was calculated using Equation 1:

$$Land - use\ cost\ (m^2 yr\ kgDW^{-1}) = \sum_u \frac{(L_a + L_f)}{DW} \qquad (1)$$

where *u* refers to each farm making up a system; L_a refers to the annual m^2 required to rear the pigs, which is the area paddocked and/or housed, excluding any land under tree cover. L_f and refers to the land required to grow feed, calculated using farm- and production stage-specific feed formulations, and quantities used of each. *DW* refers to the kg of DW produced and includes DW from finishing pigs and sows sent to slaughter, equated using economic allocation. See Methods for further explanation of method choices and data sources.

GHG cost

GHG costs included all emissions within the system boundary, shown in Supplementary Methods Figure 1 (see Methods a description of data sources).

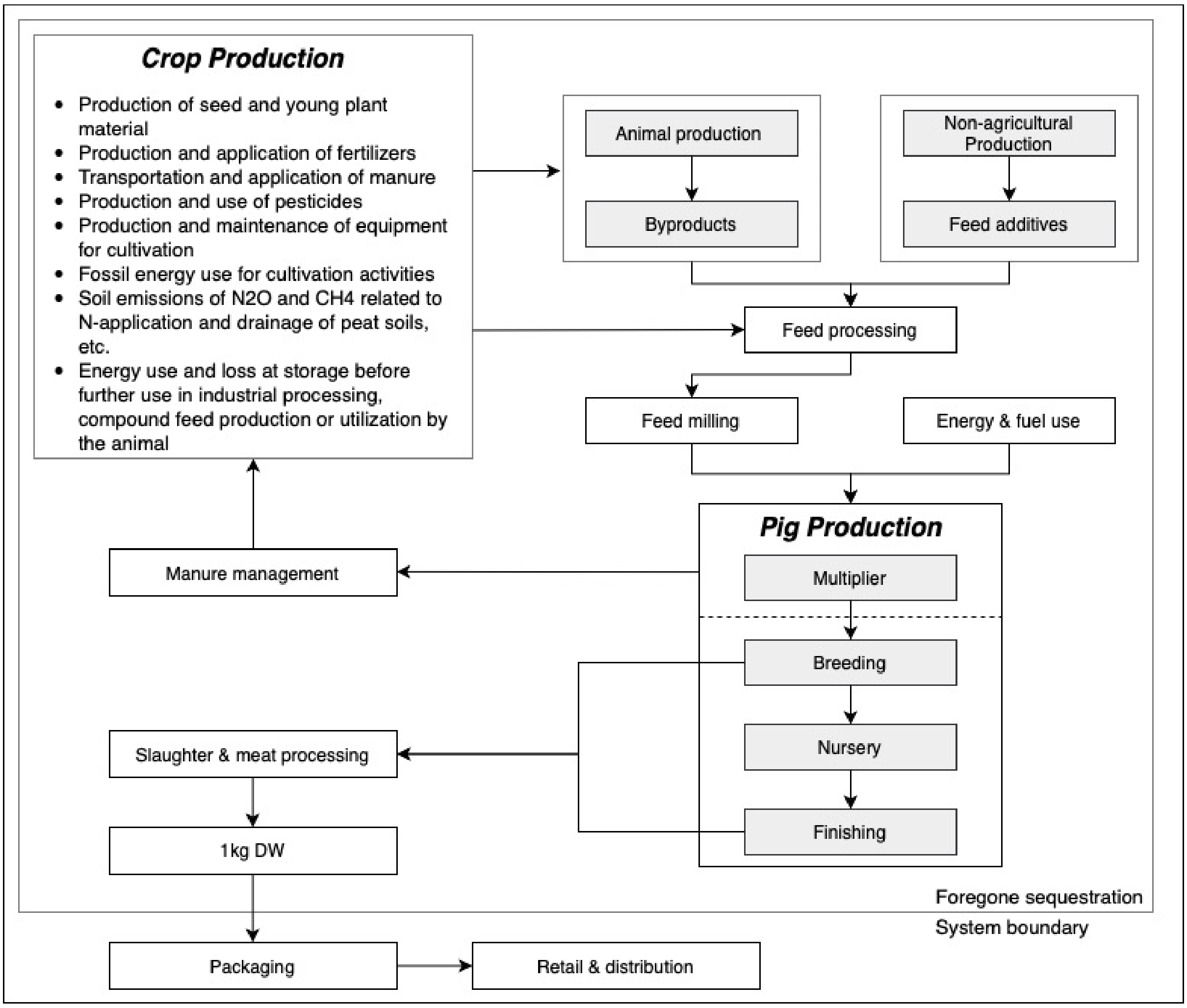

Supplementary Methods Figure 1 System boundaries for GHG cost. All transport emissions within the system boundary were included.

AMU cost

AMU cost was calculated using Equation 2.

$$AMU\ cost\ (mg\ kgDW^{-1}) = \sum_{u} \frac{AMU_{feed} + AMU_{water} + AMU_{injectable}}{DW} \quad (2)$$

where AMU_{feed} (including orally-administered antimicrobials), AMU_{water} and $AMU_{injectable}$ refer to the annual AMU in mg of active ingredient given to pigs via feed, water and injection respectively. Critically important AMU costs were also calculated in the same way, but only include European Medicines Agency[2] category B antimicrobials.

Animal-welfare cost

Animal-welfare cost was calculated using Equation 3, as in ref.[3].

$$Animal\text{-}welfare\ cost\ (quality\ of\ life\ weighted\ yr) = \sum_{i=1}^{i=4} \begin{cases} (100 - p_{iSP})w_i y_{SP} + (100 - p_{iFP})w_i y_{FP}, & p < T \\ -p_{iSP}w_i y_{SP} + -p_{iFP}w_i y_{FP}, & p \geq T \end{cases} \quad (3)$$

where *i* refers to the WQ principles of Good health, Good feeding, Appropriate behaviour and Good housing; *p* is the WQ principle score, with 0 being the worst possible score and 100 being the best; *w* is the weighting applied to each principle score; *y* is the quantity of life years needed to produce 1kg of DW, *SP* refers to sows and piglets and *FP* to fattening pigs. *T* is the WQ principle score at which a welfare cost transitions to be being a welfare benefit – where quality of life is high enough that more life-years experiencing it is deemed a benefit to animal welfare. Both *w* and *T* are challenging to quantify, but the ranking of farms and farm types was found to be largely insensitive to the choice of both[3]. Therefore, we took intermediate approaches to both: *w* was 0.35, 0.25, 0.25 and 0.15 for Good health, Good feeding, Appropriate behaviour and Good housing respectively; and *T* was 80.

References

1. Weidema, B. P. *et al.* Ecoinvent v3. vol. 3 http://www.ecoinvent.org/ (2014).

2. EMA. Categorisation of antibiotics in the European Union. *European Medicine Agency* **31**, 73 (2019).

3. Bartlett, H., Balmford, A., Holmes, M. A. & Wood, J. L. N. Advancing the quantitative characterization of farm animal welfare. *Proceedings of the Royal Society B: Biological Sciences* **290**, (2023).
