# Supplementary material for: Trade-offs in the externalities of pig production are not inevitable
Source: Nat Food. 2024 Apr 11;5(4):312–22. doi: 10.1038/s43016-024-00921-2 (PMC11045459; doi:10.1038/s43016-024-00921-2)
Supplement: Supplementary file 2 — Reporting Summary [file 43016_2024_921_MOESM2_ESM.pdf]

nature portfolio

Corresponding author(s): Dr Harriet Bartlett

Last updated by author(s): 23/03/2026

Reporting Summary

Nature Portfolio wishes to improve the reproducibility of the work that we publish. This form provides structure for consistency and transparency in reporting. For further information on Nature Portfolio policies, see our Editorial Policies and the Editorial Policy Checklist.

Statistics

For all statistical analyses, confirm that the following items are present in the figure legend, table legend, main text, or Methods section.

n/a	Confirmed	
☐	☒	The exact sample size (*n*) for each experimental group/condition, given as a discrete number and unit of measurement
☐	☒	A statement on whether measurements were taken from distinct samples or whether the same sample was measured repeatedly
☐	☒	The statistical test(s) used AND whether they are one- or two-sided<br>*Only common tests should be described solely by name; describe more complex techniques in the Methods section.*
☒	☐	A description of all covariates tested
☐	☒	A description of any assumptions or corrections, such as tests of normality and adjustment for multiple comparisons
☐	☒	A full description of the statistical parameters including central tendency (e.g. means) or other basic estimates (e.g. regression coefficient) AND variation (e.g. standard deviation) or associated estimates of uncertainty (e.g. confidence intervals)
☐	☒	For null hypothesis testing, the test statistic (e.g. *F*, *t*, *r*) with confidence intervals, effect sizes, degrees of freedom and *P* value noted<br>*Give P values as exact values whenever suitable.*
☒	☐	For Bayesian analysis, information on the choice of priors and Markov chain Monte Carlo settings
☒	☐	For hierarchical and complex designs, identification of the appropriate level for tests and full reporting of outcomes
☐	☒	Estimates of effect sizes (e.g. Cohen's *d*, Pearson's *r*), indicating how they were calculated

Our web collection on statistics for biologists contains articles on many of the points above.

Software and code

Policy information about availability of computer code

Data collection	n/a
Data analysis	We used RStudio to conduct the analysis and create the figures. All packages used are listed in the text. "Analyses were carried out in RStudio4.1.1 using the packages: “stats”, “FSA”, “ggpubr”, “rcompanion”, “ggthemes”, “patchwork” and “ggplot2"."

For manuscripts utilizing custom algorithms or software that are central to the research but not yet described in published literature, software must be made available to editors and reviewers. We strongly encourage code deposition in a community repository (e.g. GitHub). See the Nature Portfolio guidelines for submitting code & software for further information.

Data

Policy information about availability of data

All manuscripts must include a data availability statement. This statement should provide the following information, where applicable:

- Accession codes, unique identifiers, or web links for publicly available datasets
- A description of any restrictions on data availability
- For clinical datasets or third party data, please ensure that the statement adheres to our policy

Data can be found in the supplementary materials.

Human research participants

Policy information about studies involving human research participants and Sex and Gender in Research.

Reporting on sex and gender	Data on sex and gender were not collected as very few pig farmers are women and this would cause issues with retaining their anonymity.
Population characteristics	As above.
Recruitment	We contacted 150 UK pig producers, by phone or email, and 44 participated in the study. We obtained contact information for farmers from industry professionals, researchers, social media and internet searches. Sample bias was minimised by recruiting farmer types that might otherwise be under-represented with the help of industry professionals, researchers, social media and internet searches.
Ethics oversight	Ethical approval was given by the HBREC committee (application number 2018.22) at the University of Cambridge, and Plataforma Brasil prior to commencement. Before participating in the study, all farmers gave informed consent.

Note that full information on the approval of the study protocol must also be provided in the manuscript.

Field-specific reporting

Please select the one below that is the best fit for your research. If you are not sure, read the appropriate sections before making your selection.

☐ Life sciences ☐ Behavioural & social sciences ☒ Ecological, evolutionary & environmental sciences

For a reference copy of the document with all sections, see nature.com/documents/nr-reporting-summary-flat.pdf

Ecological, evolutionary & environmental sciences study design

All studies must disclose on these points even when the disclosure is negative.

Study description	We collected data from diverse pig systems representative of most commercial production systems across the world to evaluate the associations between land use, greenhouse gas emissions (GHGs), antimicrobial use (AMU) and animal welfare.
Research sample	Our sample involved pig farming systems in the UK and Brazil. We previously established an animal welfare metric based on UK farms only [Ref 34 as cited in the main manuscript]. Here, we used animal welfare data from the same farms in the UK, as well as additional data from farms in Brazil.
Sampling strategy	We contacted farmers with the help of collaborating industry professionals, internet searches and social media. Sample bias was minimised by actively recruiting farm types that might otherwise be underrepresented with the help of experts.
Data collection	H.B collected all the UK data, and H.B, M.Z, B.K, L.S, M.A and T.P collected the Brazilian data. Data were collected via a questionnaire with the farmers and on farm observations.
Timing and spatial scale	Each farm was visited once between September 2017 and December 2020. Data were collected for each farm spanning at least the most recent year.
Data exclusions	No data were excluded.
Reproducibility	n/a
Randomization	n/a
Blinding	n/a

Did the study involve field work? ☒ Yes ☐ No

Field work, collection and transport

Field conditions	Fieldwork was carried out across the UK and Brazil, and data collected spanned at least one year to control for seasonal variability.
Location	Fieldwork was carried out across the UK and Brazil. Exact locations of farms are not shared to retain anonymity.

Access & import/export	Research was approved by ethics committees at the University of Cambridge, and by Brazil's Plataforma Brasil. All data were collected in line with corresponding policies.
Disturbance	Disturbance to livestock was minimised by using animal welfare assessment methods that are not invasive and are primarily observational.

Reporting for specific materials, systems and methods

We require information from authors about some types of materials, experimental systems and methods used in many studies. Here, indicate whether each material, system or method listed is relevant to your study. If you are not sure if a list item applies to your research, read the appropriate section before selecting a response.

Materials & experimental systems

n/a	Involved in the study
☒	☐ Antibodies
☒	☐ Eukaryotic cell lines
☒	☐ Palaeontology and archaeology
☒	☐ Animals and other organisms
☒	☐ Clinical data
☒	☐ Dual use research of concern

Methods

n/a	Involved in the study
☒	☐ ChIP-seq
☒	☐ Flow cytometry
☒	☐ MRI-based neuroimaging
